# Supplementary material for: Inequities in COVID-19 vaccine and booster coverage across Massachusetts ZIP codes after the emergence of Omicron: A population-based cross-sectional study
Source: PLoS Med. 2023 Jan 31;20(1):e1004167. doi: 10.1371/journal.pmed.1004167 (PMC9888673; doi:10.1371/journal.pmed.1004167)
Supplement: S1 STROBE Checklist — (PDF) [file pmed.1004167.s001.pdf]

STROBE Statement for: "Inequities in COVID-19 vaccine and booster coverage across Massachusetts ZIP codes in Fall 2022: a population-based cross-sectional study"

Checklist of items that should be included in reports of *cross-sectional* observational studies

Section/Topic	Item No	Recommendation	Reported in [Section, Paragraph Number]
**Title and abstract**	1	(*a*) Indicate the study's design with a commonly used term in the title or the abstract	Title
		(*b*) Provide in the abstract an informative and balanced summary of what was done and what was found	Abstract
**Introduction**			
Background/rationale	2	Explain the scientific background and rationale for the investigation being reported	Background, paragraphs 1-4
Objectives	3	State specific objectives, including any prespecified hypotheses	Background, paragraph 5
**Methods**			
Study design	4	Present key elements of study design early in the paper	Background, paragraph 5
Setting	5	Describe the setting, locations, and relevant dates, including periods of recruitment, exposure, follow-up, and data collection	Methods, paragraphs 1-3
Participants	6	*Cross-sectional study*—Give the eligibility criteria, and the sources and methods of selection of participants	Methods, paragraphs 3-5, 9
Variables	7	Clearly define all outcomes, exposures, predictors, potential confounders, and effect modifiers. Give diagnostic criteria, if applicable	Methods, paragraphs 1-8
Data sources/measurement	8*	For each variable of interest, give sources of data and details of methods of assessment (measurement). Describe comparability of assessment methods if there is more than one group	Methods, paragraphs 1-9
Bias	9	Describe any efforts to address potential sources of bias	Methods, paragraphs 11-15
Study size	10	Explain how the study size was arrived at	Methods, paragraph 1
Quantitative variables	11	Explain how quantitative variables were handled in the analyses. If applicable, describe which groupings were chosen and why	Methods, paragraphs 1-10
Statistical methods	12	(*a*) Describe all statistical methods, including those used to control for confounding	Methods, paragraph 13
		(*b*) Describe any methods used to examine subgroups and interactions	Methods, paragraph 14
		(*c*) Explain how missing data were addressed	Methods, paragraph 5, 15
		(*d*) *Cross-sectional study*—If applicable, describe analytical methods taking account of sampling strategy	N/A
		(*e*) Describe any sensitivity analyses	N/A

Section/Topic	Item No	Recommendation	Reported on Page No
**Results**			
Participants	13*	(a) Report numbers of individuals at each stage of study—eg numbers potentially eligible, examined for eligibility, confirmed eligible, included in the study, completing follow-up, and analysed	Results, paragraph 1
		(b) Give reasons for non-participation at each stage	N/A
		(c) Consider use of a flow diagram	Not reported
Descriptive data	14*	(a) Give characteristics of study participants (eg demographic, clinical, social) and information on exposures and potential confounders	Results, paragraph 1, Table 1
		(b) Indicate number of participants with missing data for each variable of interest	N/A
		(c) *Cohort study*—Summarise follow-up time (eg, average and total amount)	N/A
Outcome data	15*	*Cross-sectional study*—Report numbers of outcome events or summary measures	Results, paragraph 2-3, Table 1, Fig 1, Fig 2
Main results	16	(*a*) Give unadjusted estimates and, if applicable, confounder-adjusted estimates and their precision (eg, 95% confidence interval). Make clear which confounders were adjusted for and why they were included	Results, paragraph 4-12, Table 2, Table 3, Fig 3
		(*b*) Report category boundaries when continuous variables were categorized	Results, throughout
		(*c*) If relevant, consider translating estimates of relative risk into absolute risk for a meaningful time period	All results presented as absolute risks
Other analyses	17	Report other analyses done—eg analyses of subgroups and interactions, and sensitivity analyses	Results, paragraph 11-12
**Discussion**			
Key results	18	Summarise key results with reference to study objectives	Discussion, paragraph 1
Limitations	19	Discuss limitations of the study, taking into account sources of potential bias or imprecision. Discuss both direction and magnitude of any potential bias	Discussion, paragraph 9
Interpretation	20	Give a cautious overall interpretation of results considering objectives, limitations, multiplicity of analyses, results from similar studies, and other relevant evidence	Discussion, paragraph 2-8
Generalisability	21	Discuss the generalisability (external validity) of the study results	Discussion, paragraph 9
**Other Information**			
Funding	22	Give the source of funding and the role of the funders for the present study and, if applicable, for the original study on which the present article is based	N/A
