# Supplementary material for: Inequities in COVID-19 vaccine and booster coverage across Massachusetts ZIP codes after the emergence of Omicron: A population-based cross-sectional study
Source: PLoS Med. 2023 Jan 31;20(1):e1004167. doi: 10.1371/journal.pmed.1004167 (PMC9888673; doi:10.1371/journal.pmed.1004167)

S1 Fig. Percent Vaccinated and Boosted in Massachusetts Urban Areas as of October 10, 2022

a) Percent Vaccinated

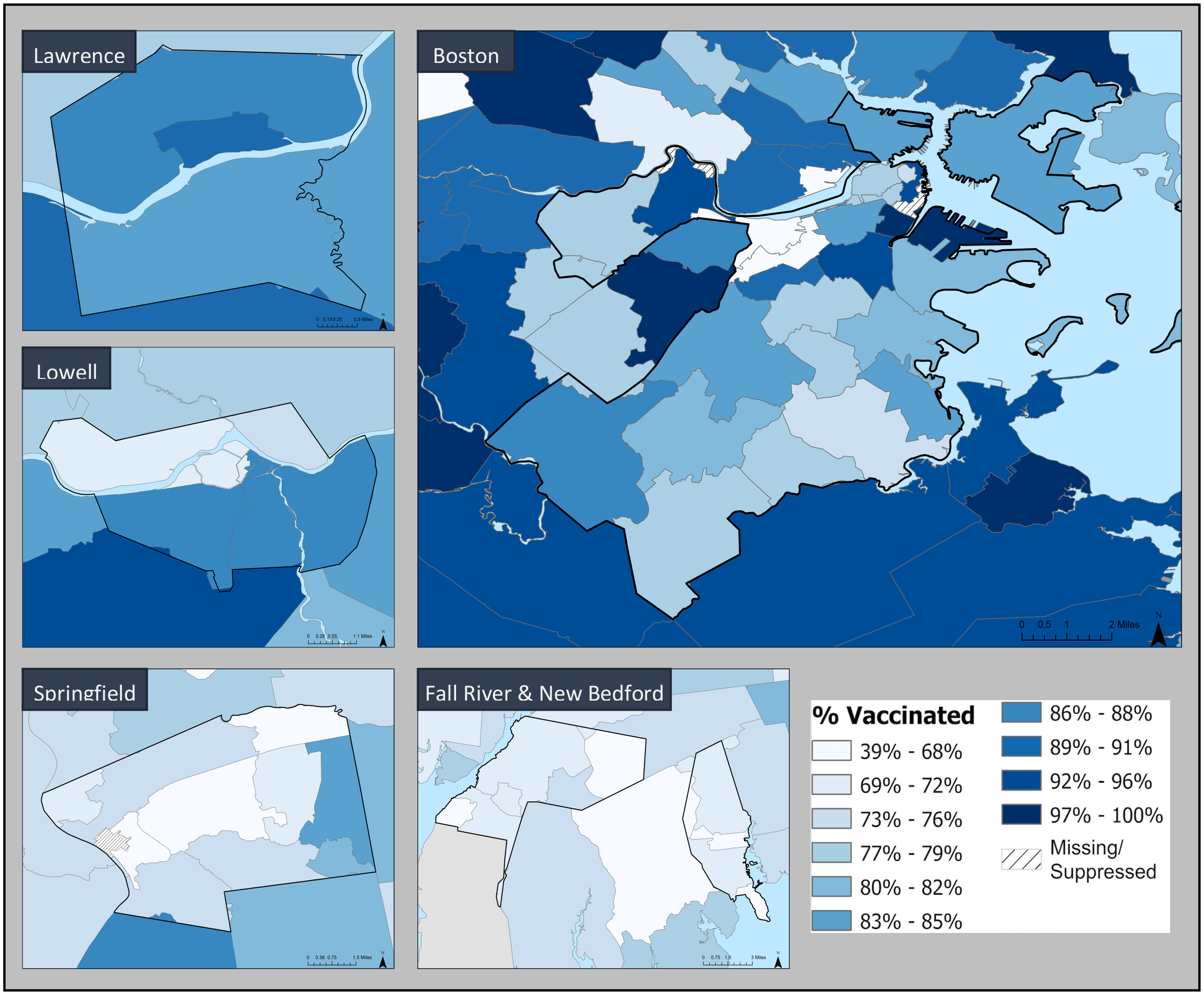

b) Percent Boosted

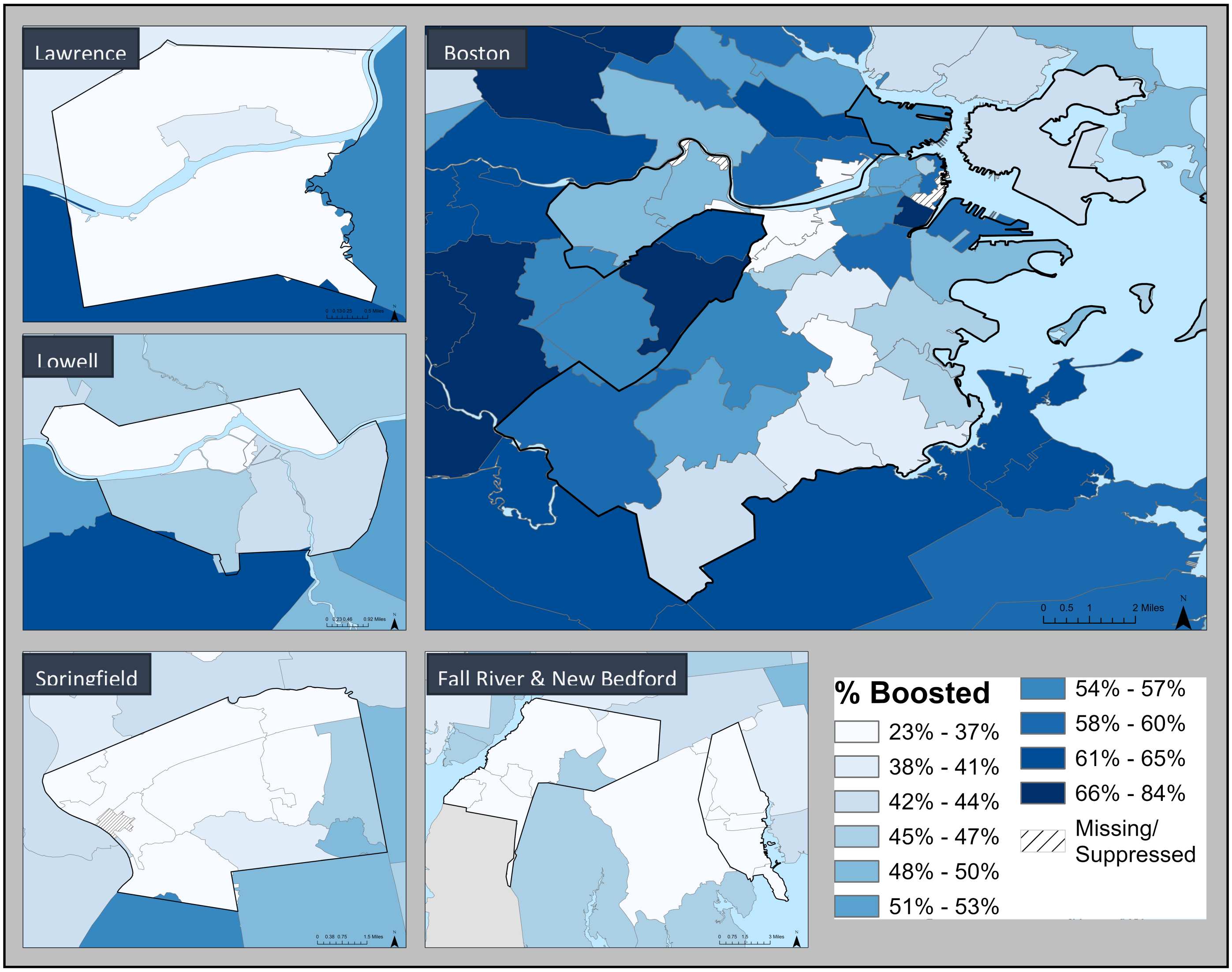

Supplement: S1 Fig — Note: S1 Fig displays higher-magnification maps of urban areas from the maps shown in Fig 1. To facilitate comparisons across ZIP codes within each panel (a) and (b), the scales in the panels differ. Shape files were obtained from the US Census Bureau, accessed October 20, 2022: https://www.census.gov/geographies/mapping-files/time-series/geo/tiger-geodatabase-file.html. Copyright protection is not available for any work of the United States Government (Title 17 U.S.C., Section 105). (PDF) [file pmed.1004167.s002.pdf]
