# Supplementary material for: Inequities in COVID-19 vaccine and booster coverage across Massachusetts ZIP codes after the emergence of Omicron: A population-based cross-sectional study
Source: PLoS Med. 2023 Jan 31;20(1):e1004167. doi: 10.1371/journal.pmed.1004167 (PMC9888673; doi:10.1371/journal.pmed.1004167)
Supplement: S1 Table — Note: Each cell shows results of a separate OLS (ordinary least squares) regression model. The crude models are bivariate. The age-adjusted include controls for ZIP code age shares in the following groups: 5–19, 20–39, 40–64, and 65+ years. Heteroskedasticity-robust 95% confidence intervals are shown in parentheses. *p < 0.05, **p < 0.01, ***p < 0.001. (PDF) [file pmed.1004167.s003.pdf]

S1 Table. Age-adjusted bivariate associations of ZIP code characteristics with percent vaccinated and boosted

**a) Vaccinated**	**Bivariate**		**Age-adjusted**	
Parameters	*beta (95% CI)*	*p-value*	*beta (95% CI)*	*p-value*
Median household income ($10k increase)	4.5*** (3.8,5.2)	<0.001	4.6*** (3.9,5.4)	<0.001
% college graduates (10 % point increase)	2.9*** (2.2,3.6)	<0.001	3.6*** (3.0,4.1)	<0.001
% Black, Latino, Indigenous (10 % point increase)	-0.8** (-1.3,-0.3)	0.002	-0.9** (-1.5,-0.3)	0.002
% essential worker (10 % point increase)	-5.5*** (-7.3,-3.8)	<0.001	-7.0*** (-8.5,-5.5)	<0.001
VEI community (0,1)	-4.0** (-6.4,-1.6)	0.001	-3.9** (-6.8,-1.0)	0.008
**b) Boosted**	**Bivariate**		**Age-adjusted**	
Median household income ($10k increase)	6.8*** (6.2,7.4)	<0.001	6.9*** (6.2,7.5)	<0.001
% college graduates (10 % point increase)	5.1*** (4.5,5.6)	<0.001	5.4*** (5.0,5.7)	<0.001
% Black, Latino, Indigenous (10 % point increase)	-2.6*** (-3.0,-2.2)	<0.001	-2.6*** (-3.2,-2.0)	<0.001
% essential worker (10 % point increase)	-11.0*** (-12.4,-9.6)	<0.001	-12.1*** (-13.2,-10.9)	<0.001
VEI community (0,1)	-11.1*** (-13.4,-8.8)	<0.001	-9.9*** (-12.9,-7.0)	<0.001

Note: Each cell shows results of a separate OLS (ordinary least squares) regression model. The crude models are bivariate. The age-adjusted include controls for ZIP code age shares in the following groups: 5-19, 20-39, 40-64, and 65+ years. Heteroskedasticity-robust 95% confidence intervals are shown in parentheses. *p<0.05, **p<0.01, ***p<0.001.
