# Supplementary material for: Inequities in COVID-19 vaccine and booster coverage across Massachusetts ZIP codes after the emergence of Omicron: A population-based cross-sectional study
Source: PLoS Med. 2023 Jan 31;20(1):e1004167. doi: 10.1371/journal.pmed.1004167 (PMC9888673; doi:10.1371/journal.pmed.1004167)
Supplement: S2 Table — Note: Each column shows results of a separate OLS (ordinary least squares) regression model. The models are stratified by sex. Each includes controls for ZIP code age shares in the following groups: 5–19, 20–39, 40–64, and 65+ years. Heteroskedasticity-robust 95% confidence intervals are shown in parentheses. *p < 0.05, **p < 0.01, ***p < 0.001. (PDF) [file pmed.1004167.s004.pdf]

S2 Table. Sex-stratified models: associations of ZIP code characteristics with percentage vaccinated and boosted

Dependent variable	Vaccinated		Boosted	
Sex	(F)	(M)	(F)	(M)
Median household income	4.57*** <0.001 (3.3,5.8)	2.97*** 0.001 (1.3,4.6)	3.48*** <0.001 (2.3,4.7)	2.24*** <0.001 (0.9,3.6)
Percent college graduates	1.84*** <0.001 (0.9,2.8)	3.76*** <0.001 (2.4,5.1)	2.70*** <0.001 (1.7,3.7)	3.56*** <0.001 (2.6,4.6)
Percent Black, Latino, Indigenous	1.24** 0.001 (0.5,2.0)	1.50*** <0.001 (0.7,2.2)	0.37 0.133 (-0.1,0.8)	0.38 0.086 (-0.1,0.8)
Percent essential worker	4.70*** <0.001 (2.6,6.8)	4.69*** <0.001 (2.2,7.2)	1.57 0.080 (-0.2,3.3)	-0.20 0.815 (-1.9,1.5)
VEI community (0,1)	-1.7 0.298 (-4.9,1.5)	-2.1 0.285 (-5.9,1.7)	-3.9** 0.004 (-6.6,-1.2)	-3.4* 0.012 (-6.1,-0.7)
R^2	0.35	0.39	0.65	0.71
N	418	418	418	418

Note: Each column shows results of a separate OLS (ordinary least squares) regression model. The models are stratified by sex. Each includes controls for ZIP code age shares in the following groups: 5-19, 20-39, 40-64, and 65+ years. Heteroskedasticity-robust 95% confidence intervals are shown in parentheses. *p<0.05, **p<0.01, ***p<0.001.
