# Supplementary material for: Inequities in COVID-19 vaccine and booster coverage across Massachusetts ZIP codes after the emergence of Omicron: A population-based cross-sectional study
Source: PLoS Med. 2023 Jan 31;20(1):e1004167. doi: 10.1371/journal.pmed.1004167 (PMC9888673; doi:10.1371/journal.pmed.1004167)
Supplement: Attachment — Submitted filename: Bor vaccines, RR1 Response to Reviewers v2.docx [file pmed.1004167.s005.docx]

October 20, 2022

Attn: Richard Turner, PhD

Senior editor, PLOS Medicine

**Re: Inequities in COVID-19 vaccine and booster coverage across Massachusetts ZIP codes**

To Dr. Turner:

Thank you for the opportunity to revise and resubmit our manuscript, “Inequities in COVID-19 vaccine and booster coverage across Massachusetts ZIP codes: a population-based cross-sectional study,” for consideration at *PLOS Medicine*.

We have attached detailed responses to the Editor’s and reviewers’ comments below. In revision, we have updated the analysis using current data (as of October 10, 2022). The findings are not substantively changed, highlighting the urgency of closing vaccination and booster inequities to protect against future COVID-19 disease.

I submit this paper on behalf of our authorship team from Boston University School of Public Health, Boston Medical Center, and the Boston Public Health Commission.

We look forward to your further feedback.

Jacob Bor ([jbor@bu.edu](mailto:jbor@bu.edu))

**Requests from the editors:**
 *1)      Please revise your title according to PLOS Medicine's style. Your title must be nondeclarative and not a question. It should begin with main concept if possible. Please place the study design in the subtitle (i.e., after a colon), e.g., a retrospective cohort study. Please place the statement, “following the winter 2022 Omicron wave” before the colon.*

We have revised the title, which now reads:

“Inequities in COVID-19 vaccine and booster coverage across Massachusetts ZIP codes in Fall 2022: a population-based cross-sectional study”

We note that we have edited the title to reflect the newly available data through October 2022.

*2)      Please include line numbers in your next draft.*

We have added line numbers.

*3)      Abstract:
a)      Please ensure that all numbers presented in the abstract are present and identical to numbers presented in the main manuscript text.*

The discrepancy arose because we were reporting “change in Y per 10-percentage point change in X” in the abstract and text and “change in Y per 1-percentage point change in X” in the tables. We believe the former is more intuitive and have now revised the tables to report changes in Y per 10 percentage point change in X, to align the numbers with the abstract and results sections.

*b)      Please mention the study design at the start of the Methods and Findings section*

We now write at the start of the Methods section: “We conducted a population-based cross-sectional study of primary-series vaccination and booster coverage 18 months into the general population vaccine rollout.”

*c)      Please quantify the main results (please present both 95% CIs and p values).*

We have added 95% CIs and p-values for all quantitative results.

*d)      In the last sentence of the Abstract Methods and Findings section, please describe the main limitation(s) of the study's methodology.*

We have added: “Limitations of the study include imprecision of the estimated population denominators, lack of individual-level demographic data, and potential for residential ZIP code misreporting in vaccination data.”

*e)      Conclusions:
i)      Please interpret the study based on the results presented in the abstract, emphasizing what is new without overstating your conclusions.*

We have rephrased our conclusions: “Eighteen months into MA’s general population vaccine rollout, there remained large inequities in COVID-19 primary series vaccine and booster coverage across MA ZIP codes, particularly among younger age groups. Disparities in vaccination coverage by racial/ethnic composition were statistically explained by differences in age and education levels, which may mediate the effects of structural racism on vaccine uptake. Efforts to increase booster coverage are needed to limit socioeconomic and racial/ethnic disparities in COVID-19 morbidity and mortality.”

*ii)     Please mention specific implications substantiated by the results.*

We now write: “Disparities in vaccination coverage by racial/ethnic composition were statistically explained by differences in age and education levels, which may mediate the effects of structural racism on vaccine uptake. Efforts to increase booster coverage are needed to limit socioeconomic and racial/ethnic disparities in COVID-19 morbidity and mortality.”

*4)      Author Summary – under the subheading, “What did the researchers do and find?” please trim the text to no more than 4 bullet points*

We have reduced the text to 4 bullet points.

*5)      Please conclude the “Introduction” with a clear description of the study question or hypothesis.*

We now write: “In this study, we assess variation in primary vaccine series and booster coverage across Massachusetts ZIP codes, analyzing data 18 months into MA’s general population vaccine rollout. Boosters are a critical line of defense in mitigating the impacts of future waves[23,24]. Although the first year of the vaccine rollout involved extensive community outreach[16], the booster campaign (including the bivalent booster that became available in September 2022) has received less public attention despite its importance for maintaining immunity and protecting against Omicron variants. Closing vaccination gaps requires an understanding of barriers to uptake and identifying geographic areas with low coverage. ZIP codes are small enough to capture socio-demographic heterogeneity that is obscured in city, town, or county-level estimates. We additionally stratified our analysis by age, the leading risk factor for severe illness due to COVID-19[25] and a strong correlate of vaccination rates. While racial disparities have often been attributed to vaccine hesitancy or medical mistrust, gaps could also result from large racial differences in income, education, and occupation that are based on structural racism and shape access to information, health services, and time off from work. We therefore assessed the extent to which socioeconomic factors explained disparities in vaccination by ZIP code racial/ethnic composition. Our analysis complements aggregate reporting by the MA Department of Public Health (DPH) on the 20 cities and towns that have been the focus of the VEI, as well as prior reports of vaccination patterns at the city/town level and across Boston ZIP codes[26].”

*6)      Did your study have a prospective protocol or analysis plan? Please state this (either way) early in the Methods section.*

We now write at the end of the FIRST paragraph of the Methods section: “The study did not have a pre-specified analysis plan.”

*a)      If a prospective analysis plan (from your funding proposal, IRB or other ethics committee submission, study protocol, or other planning document written before analyzing the data) was used in designing the study, please include the relevant prospectively written document with your revised manuscript as a Supporting Information file to be published alongside your study, and cite it in the Methods section. A legend for this file should be included at the end of your manuscript.*

N/A.

*b)      If no such document exists, please make sure that the Methods section transparently describes when analyses were planned, and when/why any data-driven changes to analyses took place.*

We now write at the end of the Methods section: “As the study did not have a pre-specified analysis plan, we describe the timing of our analytic choices here. The socioeconomic and demographic variables used in the assessment of vaccine uptake disparities were selected prior to the analysis and were chosen based on the prior literature on disparities in COVID-19 impact and early vaccine uptake. The approach to constructing population denominators followed prior work by the research team. The age ranges used in the analysis were determined *a priori* based on harmonization of ages reported in the state vaccination data and ACS denominator data. The decision to exclude ZIP codes with small populations was made after observing the high rates of data suppression and instability (wide CI’s) of estimates in those ZIP codes. The methods for the decile-based descriptive analysis and multiply-adjusted regression analyses were planned *a priori*. At the suggestion of a reviewer, we tested the null hypothesis that the VEI had the same association with vaccine coverage as with booster coverage, given that community outreach was strongest during the initial vaccine rollout.”

*c)      In either case, changes in the analysis-- including those made in response to peer review comments-- should be identified as such in the Methods section of the paper, with rationale.*

See previous response.

*7)      Please provide the name(s) of the institutional review board(s) that provided ethical exemption.*

We now write: “The Boston University Medical Campus IRB does not require ethical review for secondary analyses of publicly-available, de-identified data.”

*8)      Please ensure that the study is reported according to the STROBE and include the completed STROBE checklist as Supporting Information. Please add the following statement, or similar, to the Methods: "This study is reported as per the Strengthening the Reporting of Observational Studies in Epidemiology (STROBE) guideline (S1 Checklist)."
a)      When completing the checklist, please use section and paragraph numbers, rather than page numbers.*

We have added a STROBE statement as S1 Checklist.

We have included the statement in the Methods section: "This study is reported as per the Strengthening the Reporting of Observational Studies in Epidemiology (STROBE) guideline (S1 Checklist)."

*9)      How was race/ethnicity defined and by whom?*

We now write in the Methods section: “Race/ethnicity in the ACS was determined by self-report. People were identified as Black, Latino, or Indigenous if they reported Black or American Indian / Alaska Native among their “races” or if they reported Hispanic ethnicity.”

*10)     Please provide p values (where appropriate) in addition to 95% CIs in the main text and tables*

We have added p-values and CI’s throughout the main text and changed standard errors in the tables to CIs.

*11)     Figures and tables:
a)      Please define all abbreviations in Tables and Figures e.g., SD, HH, Blk, Hisp, Indig, OLS*

We now define all abbreviations in the notes.

*b)      Please confirm that the appropriate usage rights apply to the use of this map. Please see our guidelines for map images:* [*https://journals.plos.org/plosmedicine/s/figures#loc-maps*](https://journals.plos.org/plosmedicine/s/figures#loc-maps)

We confirm that we have usage rights. All shapefiles and maps are from the Census website and works of the United States government therefore “Copyright protection is not available for any work of the United States Government (Title 17 U.S.C., Section 105). Thus, you are free to reproduce census materials as you see fit.” See <https://www.census.gov/about/policies/citation.html>

We now write in the note to Figure 1:

“Shape files were obtained from the U.S. Census Bureau, accessed October 20, 2022: <https://www.census.gov/geographies/mapping-files/time-series/geo/tiger-geodatabase-file.html>. Copyright protection is not available for any work of the United States Government (Title 17 U.S.C., Section 105).”

c)      Please consider avoiding the use of red and green in order to make your figure more accessible to those with color blindness

We have revised the maps to use a light gray to dark blue color scale.

*12)     References:
a)      Please select the PLOS Medicine reference style in your citation manager. In-text reference call outs should be presented as follows noting the absence of spaces within the square brackets, “…population [1,2].”
b)      References should have six names before et al.
c)      Please provide a weblink for Reference #22
d)      Please ensure all weblinks are accessible
e)      Please ensure that journal name abbreviations consistently match those found in the National Center for Biotechnology Information (NCBI) databases.* [*https://journals.plos.org/plosmedicine/s/submission-guidelines#loc-references*](https://journals.plos.org/plosmedicine/s/submission-guidelines#loc-references)*.*

We have made the requested edits to the in-text citations and references list.

**Comments from the reviewers:**

*Reviewer #1: I confine my remarks to statistical aspects of this paper.  These were well done and I recommend publication.

Peter Flom*

Thank you for the review and endorsement of the methods.

Reviewer #2: Overall comments:

*This is a well-written manuscript examining inequity in vaccination and boosters in Massachusetts using aggregated state-level data. The authors use sophisticated methods to grapple with some of the discrepancies between the state-level vaccination data and population census data. The authors' present interesting and new data on associations between zip code level characteristics and vaccination and booster rates; it is an important finding to see how these disparities differ for initial vaccination and boosters.*

Thank you for this positive feedback. We have now drawn attention into the introduction the differences in outreach between the primary vaccination and booster campaign.

*Most of the comments focus on areas that could be strengthened or clarified a bit. Mostly, I think the framing of the fact that associations reverse with adjustment could be sharper…I think having this crystal clear is important so readers do not take away the "wrong" message from it.*

We appreciate the reviewer’s suggestions and have made several changes accordingly. We agree that it is important to minimize potential for misunderstanding. We now write (in the Discussion section):

“Just because disparities by racial composition are statistically explained by differences in age and education does not mean we should ignore them, or that we should infer that there are no racial disparities to address. Inclusion of race or racial composition in epidemiological analyses demands attention to underlying causal pathways.[36] Differences in education, income, and occupation between racial groups are a product of structural racism. (In MA, for example, reliance on local property taxes for education funding perpetuates schooling inequities and residential segregation.) Therefore, it is appropriate to think of these variables as mediators rather than as confounders of the relationship between racial composition and vaccine coverage.”

*I think additional exploration of the VEI program would be quite interesting…any insights into what potential impact it may have had I think would be very important to know (acknowledging there may be many limitations to being to do such an analysis).*

We agree. Unfortunately, rigorous evaluation of the VEI program is challenging for two reasons. First, the program dates to the beginning of the vaccine rollout so there’s no “pre policy / post policy” variation to study. Second, the VEI communities were basically all the “high risk” communities based on the sociodemographic variables we include in the analysis (in addition to COVID burden). It is therefore rather difficult to identify counterfactual communities. To evaluate the impact of the VEI would require comparing these “high risk” communities with other “still high but somewhat lower risk” communities, while adjusting for the factors that make communities “high risk”. This is essentially what we are doing in the regression analysis. Third, the VEI communities may have had other characteristics (e.g. limited medical infrastructure) that further hindered vaccine rollout, and which could have led to even lower vaccination rates in the absence of the initiative. Hence, interpreting differences as causal would be fraught. Fourth, qualitative evidence suggested that some VEI communities were very proactive (e.g. Chelsea, Boston) whereas others were less so, hence heterogeneous treatment effects are likely and different analyses may reveal different estimates depending on the specific source of variation. Fifth, as this is a cross-sectional analysis of current ZIP-code-level disparities, we are capturing levels of coverage, with implications for future protection, rather than timing of the rollout in different communities. Further, in the March 2022 COVID Vaccination Equity Plan, MA’s efforts to increase vaccine equity were extended beyond the original target communities to include all communities with disproportionately low vaccine coverage.

In light of these many limitations, we have decided to forgo a formal program evaluation of the VEI and to focus on current sociodemographic disparities in ZIP-code-level vaccine and booster coverage.

*Specific comments:

Intro
*       The introduction is well-written.  I think the systemic nature of root causes of the disparities could be spelled out a bit more. Many of the mechanisms of the systemic racism in healthcare are there, but they could be tied together.  Also could bring in the fact that there are different phenotypes of vaccine hesitancy/confidence, which is also rooted in disparities.*

Thank you for the opportunity to elaborate. We now emphasize multiple ways in which systemic racism has shaped vaccine coverage, including: vaccine hesitancy, rationing to the elderly and health care workers before extending access to essential workers, distribution through mass vaccination sites not in low-income communities, and pre-existing gaps in access to health information and a regular health care provider.

We now write (introduction):

“Nationally, vaccination rates are lower for less educated and lower income people and vaccine uptake has been slower in Black and Latino populations.[4,5] These populations, as well as the overlapping population of essential workers, have been at elevated risk for COVID exposure, infection, morbidity, and mortality throughout the pandemic[6–12], and inequities in vaccine coverage could further exacerbate these population health disparities.

“Vaccine hesitancy” has dominated media coverage on racial disparities, often without mention of the historical roots of medical mistrust.[13] However, other factors associated with structural racism and classism have also undermined vaccine uptake in people of color and lower-income people. When the vaccine was first rolled out, eligibility for the vaccine was limited to health care workers and then persons ages 65 and older, excluding many non-elderly, low-income, essential workers and people of color who were at high risk. Lack of access to a regular health provider and accurate health information contributed to low vaccine uptake among the uninsured, even though the vaccine was free for all who could access it. Additionally, whereas initial vaccine rollout focused on centralized mass-distributions sites, community-based delivery has since been shown to be more effective in reaching lower income people and people of color.[14,15] Lack of paid time off to get vaccinated and recover, limited outreach to non-English speakers, and low trust in the health system have also been barriers.[16,17] Broadly, race/ethnicity can serve as a surrogate for an array of socioeconomic and societal factors connected with structural racism, but few studies have interrogated these intersecting factors.”

*Methods:
*       I wasn't fully clear on the imputation approach for the neither male or female.  Did you use the ratio for the zip codes where this information was available (there were a few zip codes with this information) to impute all the rest?  Where there instances where the imputation estimated greater than 30 people for a zip code (in which case you would expect that data should not have been missing…perhaps needs a cap too).  Is neither male nor female available in census data to understand the ratio in the total population.*

The reviewer is correct about the approach. We have clarified:

We imputed data for “neither male nor female” as follows. We computed the ratios of people vaccinated/boosted among persons “neither male nor female” relative to the numbers of people vaccinated/boosted among persons either “male” or “female” for those ZIP codes reporting all three categories. We then used these ratios (0.4%, 0.3%) to estimate the number of people “neither male nor female” vaccinated/boosted based on the number either “male” and “female” for those ZIP codes that did not report numbers for “neither male nor female”. Where the estimated number “neither male nor female” exceeded the data suppression threshold of 30, we imputed a value of 30. We then aggregated across all sexes to obtain counts of ZIP code residents who had received the primary vaccine series and/or booster shot.”

The value of this ratio was 0.0038 for vaccinated and 0.0034 for boosted, reflecting that persons reporting “neither male nor female” made up around 0.4% and 0.3% of those vaccinated.

The reviewer notes that we should cap the number of persons imputed at 30, as the suppression rules indicate that the number would be observed if greater than 30. We have now implemented this cap, which is now described.

“Where the estimated number “neither male nor female” exceeded the data suppression threshold of 30, we imputed a value of 30.

In general, due to the very low numbers of people reporting “neither male nor female”, these changes have no substantive impact on our overall results for ZIP-code totals.

**       Also, do estimates change at all if you used age (I imagine this data is also fairly complete?).  I think another approach that could be considered is multiple imputation that could potentially incorporate all the reported vaccine data and zip code level characteristics.  Hopefully all of these would generate fairly similar results, but some sensitivity analyses to confirm might be nice.*

The reason we used the ZIP-by-sex data is that the ZIP-by-age data had many more cells with suppressed values. (Estimates therefore change when using the age data, in the direction of underestimating true vaccine coverage.) Thus, while we still used the ZIP-by-age data for the age-stratified analyses, we used the ZIP-by-sex data to construct ZIP-code totals, acknowledging the potential for a small degree of bias due to imputation of the “neither male nor female” category, the potential for bias is small.

**       For the zip by age analyses, it is possible to do a similar imputation process to not exclude those with missing cells.  This might require a more complicated imputation process since age distributions will likely vary across zip code level characteristics too.  Just unclear what, if any, impact this missingness has on results. Might also be helpful to present this missingness information so that extent of it is transparent.*

The ZIP-by-age file includes data on #s vaccinated and #s boosted for the following age categories: 5-11, 12-15, 16-19, 20-29, 30-39, 40-49, 50-59, 60-64, 65-69, 70-74, 75+ years. After excluding non-residential ZIP codes and the smallest ZIP codes representing 1% of the state population, there were only N=2 cells missing for # vaccinated (0.04% of all cells), and there were N=174 cells missing for # boosted (3.7% of all cells).

These are small numbers and unlikely to substantially affect the estimates. However, imputing the missing cells with “zeroes” as we have done will lead to some bias, as the true number is a value between 0 and 30.

To address this limitation, the reviewer suggests a model-based imputation approach. We now estimate mixed effects Poisson models, including fixed effects for each age group and for each age group interacted with median income, random effects for each ZIP code, and the ZIP-by-age population denominator as the offset. (Interacting age with a full set of covariates led to a model that did not converge.) We then obtain predicted counts both for the in-sample data as well as the out-of-sample, i.e. missing, cells. We find that the correlation of these predictions with the observed data is 0.99 (vax) and 0.98 (boosted).

Where data are missing for a ZIP-by-age cell, we impute by the lesser value of the model prediction and 30, the suppression threshold.

We have now implemented these changes in the revised manuscript, and describe the approach in the Methods:

“In addition to ZIP code totals, we assessed ZIP-code-by-age primary-series vaccine and booster coverage. MA DPH reports the following age categories: 5-11, 12-15, 16-19, 20-29, 30-39, 40-49, 50-59, 60-64, 65-69, 70-74, 75+ years. Some ZIP-by-age cells were missing data due to suppression rules (0.04% of cells for vaccinated, 3.7% of cells for boosted). To impute values, we estimated a mixed effects Poisson model with fixed effects for each age group and interactions of each age group with median income, ZIP-code random effects, and ACS population denominators (described below) as the offset. The model predictions were strongly correlated (0.98) with the observed values. We obtained predicted counts for the missing cells and imputed either that value or 30, whichever was smaller. We then aggregated to broader age groups for the analysis: 5-19, 20-39, 40-64, and 65+ years.”

**       The methods used to estimate percent vaccinated or boosted that accounts for the uncertainty in population estimates is sophisticated. However, I wasn't clear where the 90% confidence intervals for percent vaccinated came into play for any of the analyses.  Figures 3 to 5 seem to be using something else, but I may be mistaken.*

Figure 3 shows the distribution of point estimates and the 90% CIs for each estimate. The reason to show these estimates is to give the reader a clear picture of the uncertainty contained in ZIP-code specific estimates. While we computed these CIs for each ZIP-code, most of the paper presents decile-specific averages (Figure 4, 5) or measures of association (e.g. regression coefficients) based on these estimates (Table 2, 3). These decile averages and regression coefficients have their own 95% CIs as they are based on data that contain noise. We note that the uncertainty reflected in the 90% CIs for each ZIP code is already contained in the variance of the ZIP code point estimates and is therefore accounted for in the regression models and decile plots. By averaging across many ZIP-codes in those analyses, we reduce the noise and obtain narrower CIs.

**       I might separate out the different analyses under zip code-level and zip code by age (each its own paragraph).  Particularly for the regression and interpretation of adjustment, there is enough to warrant separating it out. I had a bit of a hard time matching them up with figures and results as I went through it.*

We have now separated the description of these analyses by paragraph in the methods section, each with its own header.

*Results
*       I wonder if by the geospatial plots it would be helpful to make the scale for vaccinated and boosted plot the same. Currently they seem to be color-coded by decile, but the most prominent finding then becomes that zip codes in the top/bottom deciles for the initial vaccine series remain there for boosters as well. Perhaps that is the point trying to be conveyed though. Either way, a figure legend that has a bit of walkthrough to highlight the main findings would be helpful.*

We went back and forth on this. The distributions of % vaccinated and % boosted are virtually non-overlapping. If we put the vaccinated and boosted on the same scale, we are essentially using half the range of the color-scale for each, hindering meaningful visualization of the variation across zip-codes for each outcome. The maps would appear largely green for %vaccinated and largely red for %boosted, however we feel that the main reason to display the maps is to show the geographic heterogeneity, rather than the difference between %vaccinated and %boosted.

We have added a note to the figure legend to clarify:

“To facilitate comparisons across ZIP codes within each panel (a) and (b), the scales in the panels differ.”

**       Figure 2 is quite informative. It shows clear relationships between zip code characteristics and vaccination (and stronger relationship for boosters). Reporting out the confidence intervals around the estimates would nice and also the p-value or correlation coeffiecient (I see that it is in Table 2 and so could also just make clear the estimates are there)*.

The reviewer correctly links the bivariate analyses in Table 2 with the decile plots in Figure 2. We now refer the reader to Table 2 in the note in Figure 2 and in the text of the results section.

“Note: fitted lines are from bivariate regressions. Coefficients and confidence intervals for bivariate and multivariable models are presented in Table 2.”

And

“Confidence intervals for these coefficients are shown in **Table 2**.”

*       *The paragraph starting with "Bivariate association…" seems like it should be included in the methods section to explain the rationale for the different regression models.*

We have moved this paragraph (with some edits) to the methods section. We now write:

“We also estimated multivariable regression models to determine whether the bivariate associations are statistically explained by other factors. For example, higher income MA residents are on average older, which could lead to a positive association between income and vaccination rates. Of particular interest, we assessed whether percent Black, Latino, and Indigenous was independently associated with vaccine coverage, after adjusting for age shares, and after adjusting for income, education, and essential worker shares – factors that may influence access to vaccination. If vaccine hesitancy related to racial/ethnic identity constrained uptake in communities of color, then we would expect to see a persistent negative association between percent Black, Latino, and Indigenous and vaccine uptake in these adjusted models.”

*       *I found Table 2 kind of hard to interpret. The reversal of the slope with adjustments seems a bit unexpected.  Is there concern for overfitting (not sure there would be with 418 zip code)?  The authors do explain this in the results and discussion, though it is still hard to align with other data in the literature (which often show independent associations of all these factors).  It may be that the in Massachusetts, the effects of systemic racism are almost fully mediated through income and education (as opposed to many other areas of the countries where this is not the case).*

Like the reviewer, we were surprised by the result. However, we believe that the findings are highly plausible and indicate, as the reviewer suggested, that income, education, and age mediate the effects of structural racism on vaccine uptake in MA.

As for the potential for overfitting, the explanatory variables – though correlated – are hardly collinear, with correlation coefficients ranging from .35 to .84 (see below). And in the regression models, we have >400 degrees of freedom. We are confident that we are not overfitting the data.

More likely is that there are contextual factors specific to MA that may contribute to these findings. We have added text to the discussion section that highlights these contextual factors and acknowledges – as a limitation – that these associations may differ in other states.

“Medical mistrust was widely noted early in the rollout, and efforts at community outreach may have been important in increasing vaccine uptake among Black, Latino, and Indigenous populations. Government engaged with community leaders in populations of color to provide information and increase interest in COVID-19 vaccination, including through the VEI. The high density of community organizations in MA facilitated diffusion of this information. Additionally, MA’s communities of color were hit hardest by the pandemic in 2020 and fear of COVID-19 was higher in these communities, e.g. resulting in higher support for remote schooling among Black MA residents. These factors might explain why demand for COVID-19 vaccination has been high in MA’s communities of color – after adjusting for age, income, and education levels. The more substantial disparities in boosters may relate to reduced outreach efforts subsequent to the initial vaccine rollout. In addition, demand for boosters could have been lower among communities and subpopulations with higher rates of infection, given the perception that acquired immunity (with or without vaccination) is sufficiently protective.

“Along with racial/ethnic patterns, substantial disparities related to income and education attainment persist, and local health departments continue to call for more long-term investments to close these gaps. Interventions to address vaccine access barriers related to education and poverty – including offering more convenient clinic times, paid sick leave for potential side effects, providing information through trusted community-based sources in multiple languages, and conducting outreach outside of the formal medical sector – will be essential to improving equity across multiple dimensions, including by race and occupation.[16]”

And in the limitations section:

“Tenth, MA is one of the wealthiest states in the country and patterns observed in MA may not hold elsewhere in the U.S.”

**       I think something else worth considering is that it may also be reflective of success of the VEI program in that it was able to attenuate many of these associations (or reverse them). In the final model, being a VEI program was associated with only 0.5% difference in vaccination…which seems very low.  Though not able to assess here, I would expect that difference to likely be higher.*

We agree and have referenced the VEI in the paragraph above as a potentially important factor in addressing prior vaccine hesitancy.

**       Ultimately, I might try to frame some of the discussion on causal interpretation thinking about mediators of racism and disparities. I always find Camara Jones work quite informative in interpreting these types of analyses/findings (*[*https://academic.oup.com/aje/article/154/4/299/61900*](https://academic.oup.com/aje/article/154/4/299/61900)*).  There are complex relationships between income, education, race, employment, and zip code and it is not as straightforward to be able to just adjust them away. Careful interpretation is required (which I think that authors do) but perhaps could go a bit deeper.*

We have added more explicit discussion on the interpretation of the results:

“Disparities by racial composition should not be ignored even if these differences can be statistically explained by socioeconomic factors. Differences in education, income, and occupation between racial groups are a product of structural racism. (In MA, for example, reliance on local property taxes for education funding perpetuates schooling inequities and residential segregation.) Therefore, it is appropriate to think of these variables as mediators rather than as confounders of the relationship between racial composition and vaccine coverage. Our analysis underscores the fact that epidemiological analyses of race/ethnicity demand attention to underlying causal pathways.[37]”

**       The impact of age structure is actually quite interesting and something I think that hasn't always been taken into account when considering raw estimates. It might also be interesting to see how this impacts the bivariate estimates too.*

We ran both raw and age-adjusted bivariate estimates. We initially suppressed the raw estimates from Table 2 to save space but have now included them as S1 Table in the supplement.

The main impact of age-adjustment is on the coefficient on median household income, which we now highlight in the text:

“Higher income MA residents are on average older, which could lead to a positive association between ZIP code income and vaccination rates”

And:

“Although median household income was strongly positively associated with vaccination and booster rates in the multivariable analyses, these associations were substantially attenuated and lost statistical significance after adjusting for age composition, suggesting that age distribution confounds the relationship between ZIP code median income and vaccination and booster rates, when controlling for other factors.”

**       I think a bivariate estimate for VEI could also be interesting. To accommodate all of the different estimates, could consider presenting all bivariate estimates in a single column.*

We have added the bivariate estimate for VEI.

We think the existing Table 2 is more accessible to the readers than putting all bivariate model coefficients into one column, but welcome the guidance of the Editors on this point.

**       Consider providing details in table 2 about coefficient interpretation (i.e., X% change per 1% increase…could also scale similarly to Figure 2).*

At the request of the Editors, we have scaled regression coefficients so that we are always reporting the same quantities in the tables, figures, and text. Specifically, we now present coefficients as “Percentage point change in Y associated with a 10-unit increase in X”, e.g. 10 percentage points or $10K in the case of median income. We have updated all numbers in the tables and text to be consistent with the figures.

We have added a note to Table 2:

“Coefficients are scaled so that they can be interpreted as the percentage point change in primary-series (or booster) coverage associated with a 10% (or $10K) increase in the continuous covariates.”

Additionally, we have listed the units under each of the predictors on the left side of the table. E.g., “Median household Income ($10K increase)”.

**       Is it possible to include case and death rates as zip code level covariates too? Not sure how readily available that data is, but is quite relevant to the targeting that happened by the VEI program it would seem.*

Unfortunately, case, death, and hospitalization data are not available at the ZIP code level, and while case data are available at the city/town level, there is a lot of important variability within cities that wouldn’t be captured.

Based on the reviewer’s suggestion, we ran a model with data on cumulative cases per capita through February 2021 at the city/town level for 351 (of 418) ZIPs that we could easily link based on city/town name between the two data files. (See table below.)

Interestingly, although higher case rates were associated with increased vaccination, none of the other coefficients changed appreciably. We have decided not to pursue this further and not to include this in the paper as the analysis was not one of our *a priori* planned analyses and the findings do not affect interpretation of results.

| *Dependent variable: Percent Vaccinated* |  |  |
| --- | --- | --- |
| *Model* | Base specification | Controlling for cases per capita  (as of Feb 2021) |
|  |  |  |
| Median household income | 1.8* | 1.3 |
|  | (0.1,3.5) | (-0.5,3.0) |
| Percent college graduates | 4.8*** | 5.3*** |
|  | (3.4,6.1) | (4.0,6.7) |
| Percent Black, Latino, Indigenous | 1.8*** | 1.8*** |
|  | (0.9,2.7) | (1.0,2.5) |
| Percent essential worker | 4.6*** | 3.4*** |
|  | (2.1,7.0) | (0.9,5.8) |
| VEI community (0,1) | -0.2 | -2.6 |
|  | (-3.5,3.1) | (-6.1,0.8) |
| COVID cases per capita |  | 75.2** |
|  |  | (29.0,121.4) |
|  |  |  |
| R^2^ | 0.35 | 0.39 |
| N | 351 | 351 |
|  |  |  |

Note: models include controls for age shares. * p<0.05, ** p<0.01, *** p<0.001

**       For Figure 3, I do know that it common enough that vaccination rates seem to exceed population estimates, though it is still odd to see. Most of the approaches to exclusion or missing data would seem to underreport vaccination rates. I am not sure if there is any strategy to address it, but could consider truncating at 100%. Would address the approaches to this in the methods.*

Some coverage estimates are above 100% because the denominators are estimated based on a random sample. While it would be possible to force the estimates to be less than 100%, such a procedure would lead to bias because we would be selectively censoring denominators with large negative error term. We have clarified in a note to the figure:

“Vertical lines are 90% confidence intervals, which capture uncertainty due to sampling error in the population denominators. Due to sampling error in the denominators, neither the point estimates nor confidence bounds are constrained to be within the [0%,100%] interval.”

We also write in the Methods section:

“We note that sampling variability in the population denominators implies that the estimated population size may sometimes be smaller than the number of residents vaccinated.”

And in the Results section:

“The point estimates contain estimation uncertainty in the denominator, which explains why some percentages exceed 100%.”

**       For Figure 4, consider putting vaccinated and boosted on same panel (and color code differently) and aligning the a-d on one page. Would also add figure legend that explains lowest to highest decile for quicker orientation for the reader.  Also consider putting children on the same figure (as opposed to figure 5).*

Great suggestions. We revised these figures accordingly and agree that the more parsimonious presentation is better. Here’s the updated version, which now puts Figs 4a, 4b, 4c, 4d, and 5 on a single panel.

**       The authors assess age strata by zip code characteristic interactions.  Is it also possible to assess race by zip code characteristic interactions also. This data seems available, though missingness may be a problem. It doesn't seem possible to essentially analyze at individual level using frequency weights, but this could be included as a limitation.*

Unfortunately, high rates of missingness imply that such an analysis would be restricted to a very narrow set of zip-codes with large populations and high percentages of Black/Latino. We have decided not to pursue this as the change in study population would make interpretation difficult.

**       Are the authors' aware of changes to the VEI efforts during the initial campaign vs. for boosters. I imagine there were. If the VEI initiative was more prominent during the initial vaccination campaign, could explore the interaction of its effect during these two periods. This is already alluded to in results for vaccination vs. booster but could be more prominent and formally assessed. Should also be included in the discussion.*

Outreach and public awareness were certainly much stronger during the initial vaccination campaign. We now mention this in the Introduction:

“Although the first year of the vaccine rollout involved extensive community outreach[15], the booster campaign has received less public attention despite its importance for maintaining immunity and protecting against omicron variants.”

Based on the reviewer’s suggestion, we undertook an additional analysis. We tested the null hypothesis that the VEI coefficient in the multiply-adjusted vaccine model was the same as the VEI coefficient in the multiply-adjusted booster model. (We estimated each model, stored the results, then used Stata’s suest command, followed by test.) The coefficient on VEI in the vaccine model was -0.3 and in the booster model is -2.1. The p-value for the null hypothesis of equal coefficients was 0.049, indicating a significant difference at the 5% level.

Our interpretation is as follows: the VEI communities may have been disadvantaged for reasons not captured by the model covariates. This disadvantage was fully mitigated by the VEI program with respects to uptake of the primary series vaccine. However, the disadvantage was not fully mitigated by the VEI program with respect to booster uptake. Although our results are consistent with lower outreach during the booster period, we do not lean too heavily on this explanation for the results because it is also possible that booster disparities could reflect later eligibility for the booster based on initial delays in vaccine access for POC populations.

We now write in the Methods: “At the suggestion of a reviewer, we tested the null hypothesis that the adjusted association between the VEI program and vaccine coverage and the adjusted association between the VEI program and booster coverage were equal.”

And in the Results: “Column (5) of **Table 2** includes an indicator for whether the ZIP code was a part of one of the 20 VEI communities targeted by the state for enhanced vaccine outreach. VEI communities had lower vaccine coverage (-4.0 percentage points, 95% CI -6.4 to -1.6, p<.01) and booster coverage (-11.1 percentage points, 95% CI -13.4 to -8.8, p<.001) than non-VEI communities, indicating that state outreach was well-targeted. After adjusting for sociodemographic characteristics and age composition, the coefficient on VEI status was close to zero: -0.3 percentage points (95% CI -3.6 to 3.0, p=.85) for vaccine coverage and -2.1 percentage points (95% CI -4.4 to 0.2, p=.079) for booster coverage. The VEI coefficients in the adjusted vaccine and booster models were significantly different (p=0.049). (We note that other factors not included in our model may explain the lower vaccination or booster rates in VEI communities, and that vaccination and booster rates might have been even lower in the absence of the VEI.)”

*Discussion

*       I think something that ends up somewhat de-emphasized in the discussion how associations changed after adjustments is that being a minority in the US and experiencing systemic racism is can't really be disaggregated from income, education, where you live, and healthcare access. The ultimate conclusions the authors reach are fine, but I think the meaning of why some of these associations attenuated after adjustment needs to be sharpened. Again Camara Jones' work is illustrative here. What comes across a bit—and I don't think the authors intended this—is that race, employment aren't the root causes and it is just income and education.*

Thank you for the opportunity to clarify our interpretation. We have revised with reference to Dr. Jones’ work.

“Far from “vaccine hesitancy”[36], these findings suggest greater demand for vaccination among populations that have been most affected by COVID, after adjusting for age and socioeconomic factors. A causal interpretation of our model would imply that ZIP codes with many essential workers or Black/Latino/Indigenous residents had lower vaccination or booster rates because their populations were on average younger, had lower levels of educational attainment and lower incomes. Access to vaccination might therefore have played an important role in the outcomes observed.

“Disparities by racial composition should not be ignored even if these differences can be statistically explained by socioeconomic factors. Differences in education, income, and occupation between racial groups are a product of structural racism. (In MA, for example, reliance on local property taxes for education funding perpetuates schooling inequities and residential segregation.) Therefore, it is appropriate to think of these variables as mediators rather than as confounders of the relationship between racial composition and vaccine coverage. Our analysis underscores the fact that epidemiological analyses of race/ethnicity demand attention to underlying causal pathways.[37]”

**       I think this finding probably needs to be contextualized in the setting of Massachusetts (I would expect the findings would differ in many other parts of the US) and perhaps also in the context of the VEI program. I am not sure it would be too far fetched to hypothesize that VEI program may be part of the reason zip codes with more minorities and essential workers outperformed expectations after adjustment specifically they were targeted for additional resources. The authors can consider whether this is reasonable or not.*

We have added some context to explain why we may have observed this result in MA.

“Medical mistrust was widely noted early in the rollout, and efforts at community outreach may have been important in increasing vaccine uptake among Black, Latino, and Indigenous populations. Government engaged with community leaders in populations of color to sensitize people to and normalize COVID-19 vaccination, including through the VEI. The high density of community organizations in MA facilitated diffusion of this information. Additionally, MA’s communities of color were hit hardest by the pandemic in 2020 and fear of COVID-19 was higher in these communities, e.g. resulting in higher support for remote schooling among Black MA residents. Based on these factors, it is unsurprising that demand for COVID-19 vaccination has been high in MA’s communities of color – after adjusting for age, income, and education levels.”

We have also added a disclaimer regarding generalizability to the limitations section:

“Tenth, MA is one of the wealthiest states in the country and patterns observed in MA may not hold elsewhere in the U.S.”

**       I think additional limitations to consider are that 1) this analysis is primarily ecological and could not use individual-level data, 2) missing data, and 3) also the discrepancy in denominators for vaccination and the census data (this is alluded to already but could be more specific).*

We have added these.

Ecological analysis:

“Sixth, we lacked individual-level data that would enable inferences on the experiences of essential workers, individuals with different income and education levels, and of different race/ethnicities. Our inferences are therefore restricted to population level statements about people in ZIP codes with different characteristics.”

Missing data:

“Third, a small percentage of MA residents did not provide ZIP codes when they received their primary series or booster vaccine. Fourth, we excluded 167 ZIP codes because they were non-residential or institutional ZIP codes, including PO Boxes, universities, and businesses with dedicated ZIP codes. Exclusion of these ZIP codes yielded a data set with greater comparability across units covering 97% of the MA population; however, some MA residents were not represented in our analysis. Due to these exclusions – particularly of university-specific ZIP codes – our totals for vaccination and booster coverage differ somewhat from published statewide estimates. Fifth, we further excluded the smallest ZIP codes (n=63) representing 1% of the population as the estimates were unstable due to very small denominators.”

Discrepancy between numerators and denominators:

“Our study has some limitations. First, our numerator and denominator data come from different sources. People may misreport their ZIP code at the vaccination site or may move to another ZIP code since receiving the vaccine. The population denominator data are estimated for ZIP codes based on aggregation of Census tract-level ACS estimates and under the assumption that the population for 2020/2021 was similar to the population for 2015-2019. Second, sampling error in the denominators results in uncertainty in coverage estimates for specific ZIP codes. Third, a small percentage of MA residents did not provide ZIP codes when they received their primary series or booster vaccine.”

**       I think in general more contextualization is needed in terms of what the findings mean beyond the different analytic strategies (e.g., this analysis was adjusted this one was not). What is larger of importance of what the results mean because of these analytic differences.* *For example, I think more contextualization is needed in terms of this finding "In fact, the higher risk of severe COVID-19 in low-income households seems to be strongly driven by personal occupational status and a foreign background." Why would this be the case? What are the mechanisms*

We have dropped that statement in revision.

We have made many revisions to put the results into context (see above) and thank the reviewer for the encouragement to be clearer in the interpretation of the underlying causal model when using racial composition in the analysis.

*Aaloke Mody*

*Reviewer #3: This is an excellent, well-written article on disparities in COVID-19 and vaccine booster uptake in Massachusetts. The authors take a careful look at racial/ethnic and other group differences in vaccine/booster uptake, adjusting for age and other factors. Among their key and important findings are that many racial/ethnic differences that were assumed to be due to differences in vaccine hesitancy are likely instead attributable to differences in age and SES distributions. The authors' framing of the issues is excellent. The methodological approaches are thoughtful and rigorously applied. The data are very timely (through March 2022). Both primary vaccine and booster uptake are examined.*

Thank you for these positive comments about the framing, methods, and timeliness of the data.

We agree that timeliness is important. In revision, we have updated our analysis through October 10, 2022, so that our data will be more useful to policy makers, given the recent roll-out of the bivalent boosters and anticipating the possibility of a surge in the Winter of 2022/2023.

Our results are nearly identical as before – with inequities if anything larger – underscoring that only modest vaccination has occurred in the past 6 months. In particular, % with a primary-series vaccine increased from 80% to 83% and % boosted increased from 43% to 50% from March 1 to October 10, 2022.

*I have only a few minor suggestions that the authors may want to consider:
1. There have been some concerns about the accuracy of vaccine coverage data from state and local health department vaccine registries. The authors mention many of the reasons why there are some limitations to the data. However, one that they don't mention is that accurately estimating vaccine coverage requires linkage of longitudinal information across individuals (i.e., dose 1, dose 2, and booster doses). If there is not good matching, this can result in underestimating or overestimating coverage. When fully vaccinated person A's dose 1 and dose 2 are not linked, it could be counted as two individuals with single doses of vaccine only. When person B and person C's first doses are incorrectly linked, then one person will be counted as fully vaccinated and not two as under vaccinated. etc. I am not sure how much of an issue this is for Massachusetts, but the authors may have some information worth sharing in the article about the data quality of the numerator. In other jurisdictions and nationally, there have been some implausible estimates of coverage by race/ethnicity, particularly on the higher end. But they may want to say more about the data generating mechanisms and data sources for the numerator data.*

This is an excellent point. Massachusetts does have a robust statewide mandatory reporting system. Massachusetts employs the Massachusetts Immunization Information System (MIIS), and all providers are mandated to report all immunizations into this system. That said, there could clearly be some issues of missingness. We now include this in the limitations section. We write:

“Ninth, it is possible that vaccine or booster coverage could be underestimated due to failure to link vaccine records longitudinally for individual residents; although all vaccinations are mandated to be reported into the Massachusetts Immunization Information System (MIIS), the foundation for the data used in our analysis, some data may be missing or incomplete, especially for those who may have received a shot outside of the state.”

*2. Could the authors clarify the source of ZIP level occupation data?*

The ZIP-code level occupation data came from the ACS. Occupation codes were designated as “essential worker” based on prior published studies conducted by the study team. We now clarify:

“Census tract level ACS 5-year estimates for 2019 were obtained for race/ethnicity (B03002), median household income (B19001), educational attainment (B15003), and occupation (S2404). Essential workers were defined based on definitions developed by the American Civil Liberties Union, which identified those job types considered “essential” during the pandemic such as work in healthcare, transportation, and food preparation[30] and has been used to assess COVID-19 inequities in MA.[10–12]”

*3. The authors mention that a key aim of the regression analysis is to determine whether bivariate associations are explained by other factors. Would it be helpful to include DAGs in the appendix, to differentiate potential confounding vs potential mediating? Or at least to discuss some of the possible reasons why bivariate associations may be fully or partially or not at all explained by other factors?*

We thank this reviewer (and reviewer 2) for flagging some confusing language in our description of the regression results. The phrase “explained by” is statistically accurate but causally imprecise, as it does not specify whether we are talking about confounders or mediators. We now make this important distinction. When we say “explained” we now write “statistically explained”. We then go on to indicate that our ecological analysis is consistent with a causal model in which income and education may mediate the causal impact of structural racism on vaccination coverage, statistically explaining racial/ethnic disparities in coverage, in addition to having their own independent impact on vaccine uptake. We further point out that age may confound the relationships between other factors and vaccine uptake due to differential COVID risk and vaccine eligibility by age group.

We now write in the Methods section:

“We also estimated multivariable regression models to determine whether the bivariate associations are statistically explained by other factors. For example, higher income MA residents are on average older, which could lead to a positive association between ZIP code income and vaccination rates. Of particular interest, we assessed whether percent Black, Latino, and Indigenous was independently associated with vaccine coverage, after adjusting for age shares, and after adjusting for income, education, and essential worker shares – factors that may influence access to vaccination. If vaccine hesitancy related to racial/ethnic identity constrained uptake in communities of color, then we would expect to see a persistent negative association between percent Black, Latino, and Indigenous and vaccine uptake in these adjusted models.”

We now write in the Results section:

“Far from “vaccine hesitancy”[36], these findings suggest greater demand for vaccination among populations that have been most affected by COVID, after adjusting for age and socioeconomic factors. A causal interpretation of our model would imply that ZIP codes with many essential workers or Black/Latino/Indigenous residents had lower vaccination or booster rates because their populations were on average younger, had lower levels of educational attainment and lower incomes. Access to vaccination might therefore have played an important role in the outcomes observed.

“Disparities by racial composition should not be ignored even if these differences can be statistically explained by socioeconomic factors. Differences in education, income, and occupation between racial groups are a product of structural racism. (In MA, for example, reliance on local property taxes for education funding perpetuates schooling inequities and residential segregation.) Therefore, it is appropriate to think of these variables as mediators rather than as confounders of the relationship between racial composition and vaccine coverage. Our analysis underscores the fact that epidemiological analyses of race/ethnicity demand attention to underlying causal pathways.[37]”

We have elected not to show a DAG because showing demographics such as age and racial composition as causal variables can be misleading.

*4. While not possible to estimate at the ZIP code level without joint distributions, it may be important for the authors to recognize the potential role of prior history of SARS-CoV-2 infection as a possible factor weighing on people's decisions to pursue a vaccine or booster. i.e., those who had covid in the past, including recent past, may be less likely to pursue a booster. And also that in considering at least the short term risks for severe outcomes during surges, it would be helpful to know the extent to which populations (say ZIP codes or by SES strata) the proportion of the population that has protection due to vaccination/boosting only or vaccination/boosting PLUS a recent history of COVID. This may also explain some of the differences between groups, including essential workers (and other groups who have endured a disproportionate share of the pandemic's burden) vs. others.*

We agree that the specific timing of surges and vaccination campaign efforts may shape who is vaccinated and who is not. We have added to the discussion section:

“In addition, demand for boosters could have been lower among communities and subpopulations with higher rates of infection, given the perception that acquired immunity (with or without vaccination) is sufficiently protective.”

*Reviewer #4: Thank you for the opportunity to review this research.

This is a well written paper.*

Thank you.

*1. For the multivariable regression Table 2a and Table 2b), how was age distribution adjusted?  Do it mean age, as a continuous variable, was included in the models?*

We have clarified in the Methods section: “We then investigated the association of ZIP code vaccination and booster coverage with ZIP code characteristics: median household income, percent college graduates, percent Black/Latino/Indigenous, percent essential worker, and percent in each age group: 5-19, 20-39, 40-64, and 65+ years.”

And we have added a note to the tables: “We adjusted for age by including variables denoting the percentage of ZIP code residents in the following age groups: 5-19, 20-39, 40-64, and 65+ years.”

*2. Figure 1: what's the difference between "missing" and "suppressed". How many zip codes were excluded because of missingness, and how many were excluded because they corresponded to specific universities or businesses, they did not correspond to residential areas, or they were assigned to post office boxes and not to residential addresses?*

We have clarified in the methods section: “We excluded ZIP codes that did not correspond to residential areas, ZIP codes that corresponded to specific universities or businesses, and ZIP codes assigned to post office boxes and not to residential addresses. This reduced the total number of ZIP codes from 648 to 481. To avoid unstable estimates due to very small denominators, we excluded the smallest ZIP codes containing 1% of the total population (n=63). After these exclusions, we had estimates of vaccination and booster coverage for all remaining ZIP codes (n=418). ”

And in the results section: “We analyzed data on 418 ZIP codes containing 97% of the MA population.”

We have added as a note to Figure 1: “Note: We analyzed data on 418 ZIP codes containing 97% of the MA population. ZIP codes labeled “missing/suppressed” were excluded because they corresponded to specific businesses or universities, to post office boxes rather than residential addresses, or because were the very smallest 1% of ZIP codes, which we excluded due to instability of estimates.”

*3. Figure 3: why the point estimate and upper bound of the confidence exceed 1? Is there any method that could make the simulated estimate less than 1?*

Some point estimates are above 1 because the denominators are estimated based on a random sample. While it would be possible to force the estimates to be less than 1, such a procedure would lead to bias because we would be selectively censoring denominators with large negative error term. We have clarified in a note to the figure:

“Vertical lines are 90% confidence intervals, which capture uncertainty due to sampling error in the population denominators. Neither the point estimates nor confidence bounds are constrained to be within the [0%,100%] interval.”

We also write in the Results section:

“The point estimates contain estimation uncertainty in the denominator, which explains why some percentages exceed 100%.”

4. Through stratified analysis, it's interesting that larger inequities in vaccination rates among younger adults and children were found. The author visually presented the difference in the inequities in vaccination rates across different age groups (Figure 4), but whether the differences are statistically significant have not been tested (Table 3).

We agree that this is a noteworthy result and have highlighted it as follows. We also now conduct tests for effect homogeneity, testing whether the coefficients on the interactions between age group and ZIP code characteristics were jointly equal to zero.

Abstract:

“Eighteen months into MA’s general population vaccine rollout, there remained large inequities in COVID-19 primary series vaccine and booster coverage across MA ZIP codes, particularly among younger age groups.”

Methods:

“To test for effect heterogeneity, we fit regression models on all age groups, interacted the covariate of interest with age, and tested the null hypothesis that the coefficients on the interaction terms were jointly equal to zero.”

Results:

**“Table 3** presents age-stratified bivariate and multivariable regression models, replicating columns (1)-(4) and (7) of **Table 2** for each age group – 5-19, 20-39, 40-64, 65+ years. Inequities in vaccine and booster uptake were most pronounced in younger age groups. In the bivariate models (panel A), income and education had positive associations with vaccine and booster coverage across all age groups, although gradient was steeper at younger ages. Percent Black, Latino, or Indigenous was negatively associated with vaccine coverage among children and younger adults and with booster coverage at all ages, but positively associated with vaccine coverage among adults age 40-64. Percent essential workers was negatively associated with vaccine coverage for non-elderly adults and children and with booster coverage for all age groups. For all ZIP code characteristics, we rejected the null hypothesis of effect homogeneity across age groups (F-test, p<0.0001).

5. *In addition to age, could sex be a potential effect modifier as well?*

Thank you for the suggestion to investigate the role of sex as a potential effect modifier. The table below shows regressions stratified by sex (similar to Table 3, by age).

In the paper, we included age as a stratifying variable (and made this decision *a priori*) because (a) age is arguably the strongest risk factor for COVID morbidity/mortality, and (b) vaccine rollout was targeted to specific age groups. While there are some potentially interesting findings in the analysis by sex (e.g. slightly different coefficients for education and income), we are inclined not to present these findings given the large set of results that we are already presenting, and as the decision to undertake this analysis was not made *a priori.*

| *Dependent variable* | **Vaccinated** | | **Boosted** | |
| --- | --- | --- | --- | --- |
| *Sex* | (F) | (M) | (F) | (M) |
|  |  |  |  |  |
| Median household income | 4.57*** | 2.97*** | 3.48*** | 2.24*** |
|  | (0.62) | (0.85) | (0.61) | (0.67) |
| Percent college graduates | 1.84*** | 3.76*** | 2.70*** | 3.56*** |
|  | (0.50) | (0.67) | (0.51) | (0.50) |
| Percent Black, Latino, Indigenous | 1.24** | 1.50*** | 0.37 | 0.38 |
|  | (0.39) | (0.38) | (0.24) | (0.22) |
| Percent essential worker | 4.70*** | 4.69*** | 1.57 | -0.20 |
|  | (1.07) | (1.27) | (0.90) | (0.85) |
| VEI community (0,1) | -17.16 | -20.79 | -39.38** | -34.47* |
|  | (16.45) | (19.42) | (13.69) | (13.74) |
| Constant | 369.94*** | 309.55*** | 201.19*** | 204.11*** |
|  | (56.26) | (65.49) | (46.21) | (43.98) |
| R^2^ | 0.35 | 0.39 | 0.65 | 0.71 |
| N | 418 | 418 | 418 | 418 |

* p<0.05, ** p<0.01, *** p<0.001
